# Degradation of Polyaromatic Hydrocarbons by Biosurfactant-Producing *Pseudomonas aeruginosa* NG4

**DOI:** 10.3390/jox15010031

**Published:** 2025-02-12

**Authors:** Shivangi Sankhyan, Prasun Kumar, Soumya Pandit, Kuldeep Sharma, Subhasree Ray

**Affiliations:** 1 Department of Life Sciences, School of Basic Sciences and Research, Sharda University, Greater Noida 201310, India; shivangisankhyan21@gmail.com (S.S.); soumya.pandit@sharda.ac.in (S.P.); 2 Department of Biotechnology, School of Engineering and Technology, Sharda University, Greater Noida 201310, India; prasun.kumar6@sharda.ac.in; 3 Centre of Research Impact and Outcome, Chitkara University Institute of Engineering and Technology, Rajpura 140401, India; kuldeep.sharma@chitkara.edu.in

**Keywords:** polyaromatic hydrocarbons, *Pseudomonas aeruginosa*, biosurfactant, biodegradation

## Abstract

Polyaromatic hydrocarbons (PAHs) are a group of organic pollutants commonly found in the environment due to industrial activities, incomplete burning of fossil fuels, and oil spills. Bioremediation of PAHs has emerged as a promising approach. This study investigated the biodegradation of PAHs (anthracene, naphthalene, phenanthrene, anthraquinone, and anthrone) at 100 ppm to 1000 ppm in the presence of glucose and glycerol by a biosurfactant-producing strain of *Pseudomonas aeruginosa* NG4. The quality of the biosurfactant produced by the bacterial strain was analyzed via emulsion index (E24), drop-collapse assay, and oil displacement assay. The PAH degradation efficiency was studied by HPLC and degradation metabolites were analyzed using GC-MS. Among all five PAHs (fed at 300 ppm), the highest degradation rates of 91.16 ± 3.64% naphthalene and 41.16 ± 1.64% anthrone were observed by *P. aeruginosa* NG4 after 10 days of incubation. The assessment of degradation intermediate metabolites revealed the PAH catabolism via the dioxygenase route, which plays a key role in the breakdown of these aromatic compounds. Biodegradation of anthrone by *P. aeruginosa* NG4 at a 300 ppm level in the media was reported for the first time. This study highlights the potential of *P. aeruginosa* NG4 as a candidate for the development of bioremediation strategies to mitigate environmental pollution caused by persistent organic pollutants like PAHs.

## 1. Introduction

Polyaromatic hydrocarbons (PAHs) are widespread pollutants produced by various human activities. The incomplete burning of fossil fuels, wood preservatives, petrochemicals, and refinery sectors primarily produces PAHs [1]. PAHs can potentially enter the environment through photochemical oxidation and volatilization, and they can also bind to organic matter found in soil and sediments [2,3]. In the United States, the Environmental Protection Agency (EPA) has classified these substances as priority pollutants because they are carcinogenic, insoluble in water, and persistent in nature [4,5]. The PAHs are resistant to breakdown due to their fused benzene ring structure and dispersed π bonds [6]. In 2004, a total of 530,000 tons of the 16 PAHs identified by USEPA were emitted into the atmosphere worldwide due to various human activities, including biofuel burning and forest fires. The contributions of both activities to the total PAH emissions were 56.7% and 17%, while the contributions of traffic fuel combustion, domestic coal combustion, and consumer products were 4.8%, 3.7%, and 6.9%, respectively. China was acknowledged as the top contributor with 114,000 tons, followed by India with 90,000 tons, the United States with 32,000 tons, and Sudan as the least recorded with 5000 tons [7]. PAH compounds are known to cause acute and chronic effects due to their high toxicity and xenobiotic and estrogenic properties [8,9]. The harmful characteristics have negative effects on various microorganisms in the marine environment, such as shellfish, fish, seabirds, marine plants, mammals, and other bottom-dwelling invertebrates [10,11]. PAH pollutants are widely dispersed throughout the atmosphere and face degradation challenges due to their stability, hydrophobicity, and polarity [12,13]. These pollutants have been found in various sources of industrial wastewater ranging from 5 to 100 mgL^−1^ [14]. However, compared to non-biological remediation techniques, especially in the prevention of soil and water pollution, the biological remediation method is commonly used for its cost-effectiveness, environment-friendly approach, wide applicability to various pollutants, and safety [15]. In addition to their effectiveness, it is important to recognize that bacteria are often exposed to a variety of mixed pollutants due to their complex characteristics [16].

The degradation of PAHs is significantly affected by their limited ability to dissolve in water, which is a major challenge. However, some microorganisms can produce a variety of surface-active compounds, commonly called biosurfactants (BSs), which can increase the availability of substrates, thereby facilitating their uptake by organisms [17,18]. BS provides an advantage in microbial bioremediation by reducing surface tension, thereby improving bacterial access to pollutants [19]. Therefore, the association between BS and the cell membrane leads to alterations that enhance the ability of the hydrocarbon to adhere to and enter the cell [20]. BS is an amphipathic compound that is produced by microorganisms such as fungi, bacteria, and yeast [17]. The chemical classification of BS includes various groups, such as lipopeptides, glycolipids, polymeric macromolecules, phospholipids, and fatty acids. Among these, the bio-based alternative surfactant rhamnolipid (a type of glycolipid) is the most effective BS [21]. Rhamnolipids are highly favored because they are biodegradable and have low toxicity [22]. Microbial treatment eliminates or stabilizes pollutants, reducing their toxicity while having minimal impact on the environment. Several bacterial species that can use PAHs as their sole source of carbon and energy have been isolated [23,24]. The majority of studies have focused on the metabolic pathways, especially those belonging to the genus *Sphingomonas*. They are used to degrade PAHs with low molecular mass, including naphthalene, phenanthrene, and anthracene, but anthraquinone and anthrone are not studied in detail. One of the most investigated bacterial species is *Pseudomonas*, which can endure harsh climatic conditions and use a wide variety of organic chemicals for growth and energy [25]. Anthrone is present in soil, air, and gases due to rising industrial activities; for instance, the combined exhaust of natural gas-fired residential space heaters have an effluent concentration of 21.4 pg∙kJ^−1^, up to 300 ng∙g^−1^ in marine sediments and 1.4 ng∙L^−1^ in water samples (https://pubchem.ncbi.nlm.nih.gov/compound/Anthrone accessed on 7 December 2024). However, anthrone degradation has not been well studied by biological routes using bacterial strains. In addition, PAH degradation in the presence of co-metabolite carbon sources has been least explored.

The objective of this study was to investigate the degradation of PAHs, including anthrone and anthraquinone, which are the least studied PAHs, along with other PAHs (anthracene, naphthalene, and phenanthrene), using the BS-producing *Pseudomonas aeruginosa* NG4. The biodegradation efficiency and underlying metabolic routes of degradation of these PAHs were studied through hyphenated analytical techniques.

## 2. Materials and Methods

### 2.1. Chemicals

All PAH compounds, including anthracene, naphthalene, phenanthrene, anthraquinone, and anthrone, were purchased from Sigma-Aldrich, St. Louis, MO, USA, and solvents such as ethanol, benzene, methanol, perdeuterated phenanthrene, and chloroform were procured from HiMedia, India. All chemicals used were of analytical grade.

### 2.2. Microorganisms and Growth Conditions

*P. aeruginosa* NG4 was previously isolated from an oil-contaminated site and identified by 16S rRNA (GenBank Accession No. OQ804633). Minimal salt medium (MSM) was used for the growth of *P. aeruginosa* NG4. The MSM broth consisted of NaCl 0.3 gL^−1^, KH_2_PO_4_ 1.4 gL^−1^, Na_2_HPO_4_ 2.2 gL^−1^, MgSO_4_⋅7H_2_O 0.6 gL^−1^, CaCl_2_ 0.02 gL^−1^, FeSO_4_⋅7H_2_O 0.01 gL^−1^, and a 0.1% solution containing trace elements: H_3_BO_3_ 0.56 gL^−1^, MnSO_4_⋅4H_2_O 1.78 gL^−1^, CuSO_4_⋅5H_2_O 1.0 gL^−1^, KI 0.66 gL^−1^, NH_4_MoO_4_⋅2H_2_O 0.39 gL^−1^, and ZnSO_4_⋅7H_2_O 2.32 gL^−1^ [19]. In each batch experiment, 100 mL of MSM medium was used and placed in a conical flask with a maximum capacity of 500 mL. The active culture of *P. aeruginosa* NG4 was also preserved at −80 °C as a glycerol stock solution for further use.

### 2.3. PAHs Biodegradation Studies

The biodegradation experiment was performed using 100 mL MSM medium with glucose and crude glycerol (CG) (1:1, %, wv^−1^, vv^−1^) as a carbon source, with a supplementation of different concentrations of anthracene, naphthalene, phenanthrene, anthraquinone, and anthrone. The MSM medium supplemented with glucose and CG (1:1, %, wv^−1^, vv^−1^) was used as a control. The concentrations of anthracene, naphthalene, phenanthrene, anthraquinone, and anthrone used in this experiment were 100, 300, 500, 750, and 1000 ppm, respectively. The overnight-grown culture (1 × 10^7^ cells mL^−1^) was inoculated to a concentration of 5% (vv^−1^) in each flask and incubated at 37 °C and 200 rpm for 10 days. The experiments were conducted in triplicate and monitored every day for growth and PAH degradation. *P. aeruginosa* NG4 growth was measured every 24 h at a wavelength of 600 nm using a UV-visible spectrophotometer to observe the optical density at various concentrations, such as 100, 300, 500, 750, and 1000 ppm.

### 2.4. Primary Characterization for BS Production

The surfactant activity of the cell-free culture produced by *P. aeruginosa* NG4 during its growth phase was qualitatively assessed by the emulsion index (E_24_), a drop-collapse assay, and an oil displacement assay, as previously described by [19].

### 2.5. Analytical Methods

#### 2.5.1. Extraction of BS

For the extraction of BS, the 10-day-old culture was centrifuged at 10,000 rpm for 20 min at 4 °C to extract the supernatant. BS was extracted using methanol and chloroform in a ratio of 1:2; the solution was mixed vigorously and left undisturbed for phase separation. The upper phase was transferred to solvent evaporation at 40 °C, resulting in the formation of a dense, solid, viscous substance known as a BS [26].

#### 2.5.2. High-Performance Liquid Chromatography (HPLC)

HPLC was performed to determine the residual concentrations of PAHs. After the biodegradation process, the residual PAHs were extracted using an equivalent volume of ethyl acetate. Subsequently, each sample was collected and transferred into a 2 mL tube. Following this, the tube was filled with 1 mL of ethyl acetate and allowed to dry at room temperature. Subsequently, 1 mL of methanol was used to dissolve the dried PAH residues and then analyzed using an HPLC system equipped with an Atlantis dC18 5 μm column (Waters, Breeze QS HPLC, Milford, MA, USA). The mobile phase, which consisted of methanol and water in a ratio of 90:10 (vv^−1^), was used at a flow rate of 1 mLmin^−1^, and 20 μL was injected for the analysis of residual PAHs [27]. The analysis of PAH degradation was conducted after 10 days. The calibration curve for naphthalene was prepared at concentrations of 20–1200 µg/mL, and for other PAH compounds, these concentrations were 2–50 µg/mL. Calibration curves were produced by averaging the peak regions of each compound on the chromatograms acquired from three injections, with measurements at a wavelength of 261 nm.

#### 2.5.3. Gas Chromatography–Mass Spectroscopy (GC-MS) Analysis

The metabolites of PAHs’ degradation pathways were identified using GC-MS analysis. After a cultivation period of 10 days, the PAHs were extracted using 100 mL of ethyl acetate. From each sample, the organic phases were mixed, and 1 mL of ethyl acetate was transferred to a 2 mL tube and dried at room temperature. The dried residue was dissolved in 1 mL of methanol and examined using GC-MS (Agilent 8890/5977B, Agilent Technologies, Santa Clara, CA, USA) [27]. For analysis, an HP-5 MS column (30 × 0.32 mm) was used for separation, and electron impact mode was used for detection at 70 eV. Helium was used as the carrier gas, flowing through the column at a rate of 1.0 mLmin^−1^. The column was kept at 290 °C, and 20 μL of the extracted sample was injected into it. Perdeuterated phenanthrene was used as an internal standard at 10 mg/L in hexane [28].

### 2.6. Antibiotic Sensitivity Detection of P. aeruginosa NG4

The antibiotic sensitivity of the selected strains was evaluated using the Kirby–Bauer disk diffusion method on Luria–Bertani (LB) agar, with commercially available antibiotic discs of ampicillin (10 µg), streptomycin (10 µg), vancomycin (30 µg), tetracycline (30 µg), and chloramphenicol (30 µg). The overnight-grown culture was spread on an LB agar plate. However, sterile forceps were used to place the antibiotic discs, and the plates were incubated for 24 h at 37 °C. After incubation, a distinct zone of inhibition was observed around each antibiotic disc and measured in centimeters (cm) [29]. All experiments were performed in triplicate.

### 2.7. Statistical Analysis

Based on three independent replicates (*n* = 3), the data are displayed as mean ± standard deviation (SD). GraphPad InStat 3.10 was used to analyze the data. One-way analysis of variance (ANOVA) was performed to evaluate significant differences between multiple data sets, with a significance level of *p* < 0.05 [30]. All experiments were performed in triplicate.

## 3. Results

### 3.1. Growth Profile

**Figure 1:**
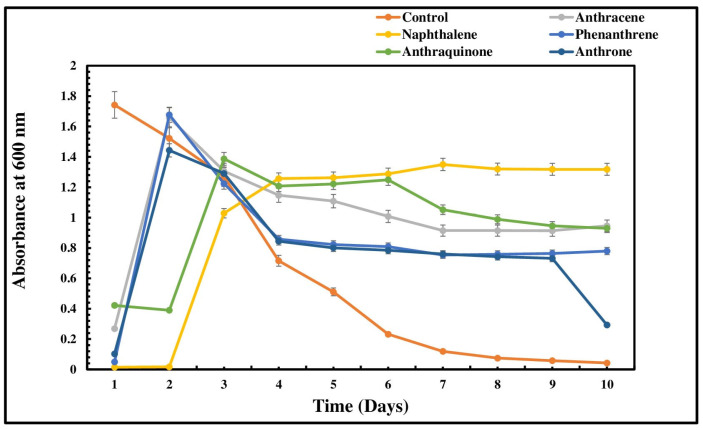
Growth profile of *P. aeruginosa* NG4 at 300 ppm of PAH supplementation.

The growth profile of *P. aeruginosa* NG4 was studied using MSM medium supplemented with glucose and CG (1:1, %, wv^−1^, vv^−1^) along with different concentrations of anthracene, naphthalene, phenanthrene, anthraquinone, and anthrone ranging from 100 to 1000 ppm, respectively. MSM medium supplemented with glucose and CG (1:1, %, wv^−1^, vv^−1^) was used as a control. The culture of *P. aeruginosa* NG4 grown separately with five PAH compounds—anthracene, naphthalene, phenanthrene, anthraquinone, and anthrone—showed similar growth patterns, with a lag period of about 2–3 days. There was a sharp increase in the bacterial growth at 3–4 days of incubation just after the lag period, indicating the culture’s acclimatization (Figure 1). After initial stimulation, some stabilization of growth was observed with a gradual but steady decline in the bacterial growth.

### 3.2. Degradation Study of PAHs by P. aeruginosa NG4

The biodegradation of all five PAH compounds by *P. aeruginosa* NG4 was studied for a period of 10 days. The residual PAH levels were analyzed by HPLC to check the degradation efficiency. Samples from all PAH doses (100, 300, 500, 750, and 1000 ppm) showed degradation to a varying range, with the final value obtained after 10 days of incubation, as shown in Table 1. The biodegradation study of anthracene by *P. aeruginosa* NG4 showed that the degradation increased from 72.12 ± 2.03% to 88.86 ± 3.55% at 100 to 300 ppm, respectively. However, at higher concentrations of anthracene, the degradation efficiency decreased to 36.19 ± 0.25%. Similarly, 82.18 ± 3.28% naphthalene degradation was observed at a concentration of 100 ppm within 10 days. Increasing the naphthalene level to 300 ppm resulted in 91.16 ± 3.64% degradation as calculated by the respective standards using HPLC (Figure S1). Further increasing the level led to a steady decline in the degradations at 500 ppm, the degradation was 80.19 ± 3.20%, which declined further to a level of 64.41 ± 1.97% at 1000 ppm. Phenanthrene degradation showed similar behavior, wherein the degradation level increased up to 300 ppm and then declined gradually to 57.55 ± 1.31% at 1000 ppm. In contrast, the biodegradation studies for anthrone demonstrated that at a concentration of 100 ppm, 30.56 ± 1.22% was degraded within 10 days. At 300 ppm, 41.16 ± 1.64% was degraded; 22.17 ± 0.88% was degraded at 500 ppm; 13.99 ± 0.55% anthrone was degraded at 750 ppm; and at a concentration of 1000 ppm, 8.33 ± 0.13% was degraded by *P. aeruginosa* NG4. For anthraquinone, the biodegradation studies revealed a similar pattern, though the level of degradation was lower even at 100 ppm, and a maximum of 32.06 ± 1.28% degradation was observed after 10 days of incubation. At 300 ppm, 33.82 ± 1.35% was degraded, which was reduced to 21.51 ± 0.86% at 750 ppm. At a concentration of 1000 ppm, 15.40 ± 0.23% anthraquinone was degraded by *P. aeruginosa* NG4.

### 3.3. Characterization for BS Production

*P. aeruginosa* NG4 culture was qualitatively analyzed for BS production using a culture supernatant via oil displacement, drop collapse, and emulsification index measurement, as shown in Figure S2. The surface activity of the culture supernatant produced a zone of clearance, indicating the presence of BS. Higher clearance of engine oil through the supernatant reflects higher surfactant activity. Based on the observation, it is imperative that the strain produce a significant amount of BS in the presence of PAHs. A similar observation was recorded with the drop collapse assay for all the experimental conditions, suggesting BS production. However, the assay with anthraquinone showed a low potential for drop collapse compared to others. The emulsification index is an indirect parameter for the surfactant ability of any compound. Here, a varying amount of emulsification was observed with the culture supernatant in all the experimental conditions. A maximum emulsification activity (E_24_) was observed in the 10-day-old culture (cell-free supernatant) grown in the presence of naphthalene at 88.23% against engine oil at 1000 ppm. These findings show that *P. aeruginosa* NG4 strains are capable of producing BS.

### 3.4. Identification of PAH Degradation Metabolites by GC-MS Analysis

**Figure 2:**
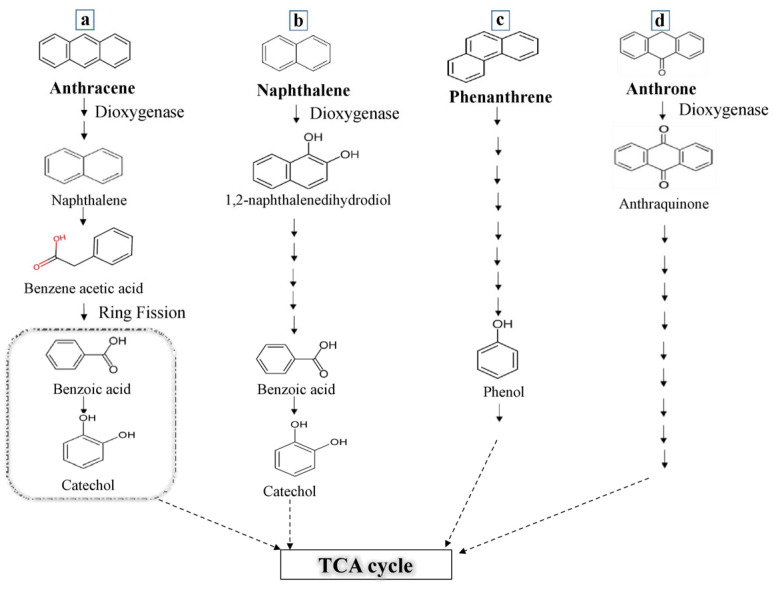
Proposed degradation pathway by *P. aeruginosa* NG4 based on the metabolites’ degraded products obtained from (**a**) Anthracene, (**b**) Naphthalene, (**c**) Phenanthrene, and (**d**) Anthrone.

The PAH degradation potential of *P. aeruginosa* NG4 was evaluated through GC-MS analysis. The metabolites identified from degradation of PAH compounds by *P. aeruginosa* NG4 at 300 ppm are shown in Table 2. The anthracene degradation released intermediate derivatives such as (i) 2-Amino-1-naphthol, (ii) N-(2,6-dimethylphenyl)-2-ethoxy-5-(tetrazol-1-yl)-benzenesulfonamide, (iii) benzoic acid, and (iv) Catechol. The primarily derivative compounds of anthracene were benzoic acid, naphthalene, and catechol, which are major degradation products, as shown in Figure 2. On the other hand, the derivative compounds observed during naphthalene degradation included: (i) naphthalene, 1,8 Deutero, (ii) catechol, (iii) Naphtho [1,2-c] thiophene, 1,3-dihydro-, 2-oxide, (iv) Naphthalene,1-isocyano, and (v) benzoic acid. The derivative compounds of naphthalene included benzoic acid and catechol. The degradation of naphthalene was dependent on the type of dioxygenase enzyme present in the bacteria. Similarly, *Pseudomonas* spp. are known to possess dioxygenase enzymes that degrade naphthalene and convert it into 1,2-naphthalenedihydrodiol. The latter is further metabolized to produce benzoic acid, which in turn converts to catechol and is subsequently mineralized by entering the TCA cycle. Similarly, the degradation compounds of phenanthrene included (i) 2,4-Di-tert-butylphenol and (ii) Tris (2,4-di-tert-butylphenyl) phosphite. As mentioned earlier, through the same degradation pathways, phenanthrene is converted into phenol and enters the TCA cycle (Figure 2). The degradation products detected from anthraquinone were (i) N-(2,6-dimethylphenyl)-2-ethoxy-5(tetrazol-1-yl)-benzenesulfonic acid and (ii) 2-Bromo-N-[(1-(cyclohexylmethyl)-1H-1,2,3-triazol-4-yl) methyl]-N-methylbenzenesulfonic acid. It has been reported earlier that, by *Sphingomonas* sp., anthraquinone is first converted into 1-amino-4-bromoanthraquinone-2-sulfonic acid, undergoes hydrolyzation, and forms two compounds, i.e., 2-amino-3-hydroxy-5-bromobenzenesulfonic acid and 2-amino-4-hydroxy-5-bromo- benzenesulfonic acid [31]. As compared to the previous study, this study identified benzenesulfonic acid as the major degraded metabolite of anthraquinone by *P. aeruginosa* NG4. Meanwhile, (i) anthraquinone and (ii) 1,4-Anthraquinone were obtained as the major degradation products of anthrone.

## 4. Discussion

PAH are the hazardous compounds, and BS plays an important role in the degradation of PAH by increasing their bioavailability and facilitating their uptake by microorganisms. In the previous study, co-utilization of glucose and CG (1:1, %, wv^−1^, vv^−1^) at 37 °C for 4 days was found to be a favorable approach for the production of BS [17]. The present study investigates the degradation of PAHs by the BS-producing strain *P. aeruginosa* NG4 over an extended period of 10 days. The study found that *P. aeruginosa* NG4 produced BS when PAH and glucose and CG (1:1, %, wv^−1^, vv^−1^) were given as the sole carbon source and demonstrated an increased degradation rate of PAHs. These findings suggest that the production of BS by *P. aeruginosa* NG4 significantly contributes to the degradation of PAHs. An observation of microbial growth compared to the control over a period of 10 days of incubation suggested that the PAH compounds did not severely inhibit growth. Indeed, the microbial culture of *P. aeruginosa* NG4 was able to tolerate a higher PAH concentration of 1000 ppm and metabolize them for their growth. *P. aeruginosa* has been reported to degrade PAH compounds such as anthracene, naphthalene, phenanthrene, and anthraquinone [32]. However, anthrone degradation by *Pseudomonas* spp. has not yet been reported. The biodegradation order for all five PAH compounds by *P. aeruginosa* NG4 was observed as follows: naphthalene > anthracene > phenanthrene > anthrone > anthraquinone. Thus, the result suggests that *P. aeruginosa* NG4 exhibited the highest degradation of naphthalene at 300 ppm, with 91.16 ± 3.64%. On the basis of one-way ANOVA, the PAH concentration of 300 ppm was found to be optimal for the degradation by *P. aeruginosa* NG4 with a *p*-value of <0.05 (Table 1). Therefore, due to BS and the availability of naphthalene to cells, its solubility increases relative to its saturation level. For the other PAHs, the maximum degradation was also achieved at 300 ppm, showing >80% degradation (Table 1). However, as the concentration increased to 500, 750, and 1000 ppm, the residual amount of PAHs increased, indicating a decline in degradation efficiency. Despite the unaffected growth of *P. aeruginosa* NG4 at higher concentrations, the efficiency of PAH degradation decreased significantly at higher concentrations. A similar observation was made earlier with *Pseudomonas* isolate grown on Tanner’s Mineral Medium using PAHs as the sole carbon source [33]. In the previous study, 80% of phenanthrene was degraded by *P. aeruginosa* strain W10 at a concentration of 200 ppm for 30 days [18]. Another Gram-negative bacterium, *Burkholderia cepacia* 2A-12, could tolerate up to 800 ppm of PAHs and mineralize phenanthrene and naphthalene (as a sole carbon source), but not pyrene [34]. Degradation of more than one PAH was also reported using *P. aeruginosa* at a concentration of 250 ppm to 750 ppm, where it was observed that naphthalene was completely degraded within 10 days, while the bacterial strain took 14 days to completely metabolize anthracene [33]. Similar to Gram-negative microorganisms, few Gram-positive bacterial strains are known to efficiently metabolize naphthalene. For instance, *Streptomyces* sp. AB1, *Streptomyces* sp. AH4, and *Streptomyces* sp. AM2 have been reported to degrade up to 85.23% naphthalene after 12 days of incubation [35]. *B. licheniformis* MTCC 5514 can degrade 95% of anthracene at a concentration of 300 ppm [25]. The polyaromatic compounds are widely distributed in nature, including pigments and dyes. In a previous study, *Pseudomonas* strain GM3 was found to degrade up to 24% of Reactive Blue 2 and up to 84% of Acid Green 27 (anthraquinone-dyes) within 48 h when fed with nutrient-rich media and 100 ppm of respective dyes [31]. This reflects the ability of *Pseudomonas spp*. to mineralize PAH compounds and their potential use in bioremediation. The increased degradation rates against the PAHs by *P. aeruginosa* NG4 may also be attributed to its ability to produce BS, which increases the solubility of hydrophobic PAHs and facilitates their breakdown. Although the *P. aeruginosa* strains have the potential for biodegradation, other microbes may also have the untapped ability to degrade PAHs in a synergistic manner [33]. It would, thus, be imperative to study a microbial consortium involving *P. aeruginosa* NG4 as one of the major contributors to cooperative interactions arising from distinct complementary biochemical PAH degradation pathways within different strains. It has been observed that within a consortium, the removal of PAH metabolites by one strain that may otherwise inhibit the activity of other strains occurs through cooperative interactions. In such cases, as a major constituent of consortia, *P. aeruginosa* NG4 may prove helpful—firstly, the BS produced by the strain will be helpful in the assimilation of PAHs into the cell membrane and modifications in cell hydrophobicity; secondly, the BS may be utilized as a carbon source, leading to a substantial biomass increase. However, it will require further study to understand the role of the BS-producing *P. aeruginosa* strain in PAH degradation. Interestingly, there are studies that have shown that an individual strain of *P. aeruginosa* is equally capable of degrading anthracene and phenanthrene as it is a mixed microbial consortium. However, the difference in the degradation was in the order of naphthalene > anthracene, corresponding to the observation made in this study. Such a degradation rate is plausibly due to the linear two-ring structure of naphthalene compared to the three-ring structure of anthracene, although the initial attack of the microbial enzymes occurs in the K- and bay-regions of the compound [33]. In the previous study, naphthalene and benzeneacetic acid were the major degradation products detected from anthracene by *B. licheniformis* MTCC 5514 [25]. A detail of PAH biodegradation by a single bacterial strain is shown in Table 3. As compared to previous studies, this study has identified most of the degraded intermediary metabolites of anthracene released during the 10-day incubation of *P. aeruginosa* NG4. In an earlier study, benzaldehyde, benzoic acid, and catechol were identified as the primary intermediates produced by *Streptomyces* spp., while the degradation of naphthalene by thermophilic bacterium *B. thermoleovorans* generated phthalic acid, benzoic acid, and salicylic acid as the major intermediate metabolites [28,36]. *P. anguilliseptica* Strain-A1 produced 9,10-phenanthrenequinone as one of the major degraded products [37]. As compared to previous studies, the present study shows that phenol is an intermediate metabolite in the degradation of phenanthrene by *P. aeruginosa* NG4. In this study, anthraquinone was identified as the primary degraded metabolite of anthrone by *P. aeruginosa* NG4. As anthrone is a derivative of anthracene, which forms during the oxidation process, its degradation may have the same fate (Figure 2) [38]. In addition, several *Pseudomonas* species, including *P. alcaligenes* PA-10, *P. aeruginosa* DQ8, *P. fluorescens* ACB, and *P. putida* NCIB 9816, were reported to break down PAHs such as phenanthrene, naphthalene, pyrene, anthracene, chrysene, and fluoranthene. Most of them were found to secrete dioxygenase enzymes, which play important roles in initiating the degradation of PAHs [39]. Intracellular enzymes, as well as bacteria belonging to another genus (in the case of a consortium), were found to be responsible for further breaking down the initial degradation products. As a result, complete degradation of PAHs depends on the activity of both extracellular and intracellular enzymes of the microbial strain [39]. In order to survive in the environment, bacteria produce and degrade various compounds, including antibiotics, for competitive advantages. Members of this *Pseudomonas* genus have a remarkable ability to degrade a plethora of chemicals, aromatic hydrocarbons, metals, metalloids, and many other recalcitrant pollutants. For bioremediation applications and survival in the field, it is desirable to be aware of the resistance and sensitivity patterns of microorganisms (Figure S3). The resistance to ampicillin of the strain NG4 (Table S1) could be attributed to the fact that this antibiotic is frequently obtained in the environment, encouraging the evolution of resistance by autochthonous strains. Thus, such a survival strategy may allow them to proliferate ahead of other microbes in the community. Moreover, the metabolic versatility is an indication of the occurrence of multifunctional dioxygenases in *P. aeruginosa* NG4 [40]. In addition, the strain used in this study is sensitive to first-generation antibiotics, except ampicillin (Table S1, Figure S3). This reflects that the use of this strain is unlikely to cause human health issues. However, *P. aeruginosa* is classified as an RG-2 strain, which reflects that it is an opportunistic pathogen that rarely causes disease in immune-compromised humans or animals, and is unlikely to be a serious health hazard. *P. aeruginosa* is naturally found in the environment, including in soil, food, water, and other habitats, at varying CFU levels [41]. Compared to other pathogens, this Gram-negative bacterium does not form spores, so the population remains in check due to natural mechanisms, as it only grows in humid environments. Due to its ubiquitous nature, it is impractical and unproductive to avoid their contact with us via food, water, etc. Moreover, there have been quite a few studies on biological degreasing systems which use bacteria and health risk assessments of workers that have revealed no health issues under standard operating conditions [42].

## 5. Conclusions

In this study, *P. aeruginosa* NG4 was used for the degradation of PAH compounds such as anthracene, naphthalene, phenanthrene, anthraquinone, and anthrone with the supplementation of simple carbon sources. The results showed that, at a concentration of 300 ppm, 88.86 ± 3.55% of anthracene, 91.16 ± 3.64% of naphthalene, 80.56 ± 3.22% of phenanthrene, 33.82 ± 1.35% of anthraquinone, and 41.16 ± 1.64% of anthrone were degraded by *P. aeruginosa* NG4. Among all five PAHs, naphthalene showed the highest rate of degradation after 10 days of incubation, followed by anthracene. Interestingly, anthrone degradation of up to 41.16% was observed. This study demonstrates that *P. aeruginosa* NG4 is an effective PAH degrader, and the bioavailability of PAHs may be attributed to the emulsification abilities of BS produced by this strain. The assessment of intermediate metabolites revealed PAH degradation via the dioxygenase route. Thus, it is apparent that *P. aeruginosa* NG4 has the potential to tolerate up to 1000 ppm of PAH. The bacterial strain can effectively metabolize 300 ppm of PAHs within 10 days of incubation. Therefore, this strain can be exploited to significantly reduce or eliminate hazardous compounds from wastewater and industrial effluents containing PAH and PAH-based dyes. In addition, to the best of our knowledge, this is the first report demonstrating anthrone degradation by *P. aeruginosa*.

## Figures and Tables

**Table 1:** Degradation of polyaromatic hydrocarbons by BS-producing *P. aeruginosa* NG4.

Polyaromatic Hydrocarbons	*P. aeruginosa* NG4 Grown on MSM Medium and Glucose: Glycerol (1:1, wv^−1^, 1% vv^−1^) Supplemented with PAHs				
	100 ppm	300 ppm	500 ppm	750 ppm	1000 ppm
	Degradation Level (%)				
Anthracene	72.12 ± 2.03	88.86 ± 3.55	79.41 ± 3.05	67.18 ± 2.68	36.19 ± 0.25
Naphthalene	82.18 ± 3.28	91.16 ± 3.64	80.19 ± 3.20	78.96 ± 3.15	64.41 ± 1.97
Phenanthrene	78.12 ± 3.12	80.56 ± 3.22	70.60 ± 2.82	69.90 ± 2.79	57.55 ± 1.31
Anthraquinone	32.06 ± 1.28	33.82 ± 1.35	30.08 ± 1.20	21.51 ± 0.86	15.40 ± 0.23
Anthrone	30.56 ± 1.22	41.16 ± 1.64	22.17 ± 0.88	13.99 ± 0.55	8.33 ± 0.13

**Table 2:** GC-MS analysis of intermediary metabolites generated during the degradation of PAHs produced by *P. aeruginosa* NG4.

Retention Time (min.)	Area	Compound Name
Anthracene		
14.456	652779	2-Amino-1-naphthol
16.189	515237	N-(2,6-dimethylphenyl)-2-ethoxy-5-(tetrazol-1-yl)-benzenesulfonamide
20.183	2220508	Benzoic acid
20.921	6657002	Catechol
Naphthalene		
14.478	756300	Naphthalene, 1,8 Deutero
16.132	559660	Catechol
20.212	6965756	Naphtho [1,2-c] thiophene, 1,3-dihydro-, 2-oxide
20.944	21463719	Naphthalene,1-isocyano
22.695	121583	Benzoic acid
Phenanthrene		
12.407	482529	2,4-Di-tert-butylphenol
25.287	571302	Tris(2,4-di-tert-butylphenyl) phosphite
Anthraquinone		
16.235	231391	N-(2,6-dimethylphenyl)-2-ethoxy-5-(tetrazol-1-yl)-benzenesulfonic acid
22.878	75130	2-Bromo-N-[(1-(cyclohexylmethyl)-1H-1,2,3-triazol-4-yl) methyl]-N-methylbenzenesulfonic acid
Anthrone		
17.694	1461581	Anthraquinone
		1,4-Anthraquinone

**Table 3:** PAHs biodegradation efficiency using a single bacterial strain.

Microorganism	PAH Compound	Incubation Period (Days)	Test Range (ppm)	% Biodegradation Efficiency (Maximum Degradation)	Reference
*Pseudomonas stutzeri* BUK_BTEG1	Naphthalene	3	100–1000	85.58 (800 ppm)	[23]
*Streptomyces* sp. ERI-CPDA-1	Naphthalene	7	100	99.14 (100 ppm)	[36]
	Phenanthrene			17.5 (100 ppm)	
*P. aeruginosa* W10	Phenanthrene	30	200	80 (200 ppm)	[18]
*Streptomyces* spp. AB1, AH4, and AM2	Naphthalene	12	100	82.36, 85.23 and 81.03 (100 ppm)	[35]
*P. aeruginosa*	Naphthalene	10	250–750	99 (250 ppm)	[33]
	Anthracene			96 (250 ppm)	
*Aeribacillus* sp. (UCPS2)	Anthracene	15	200	92–96 (200 ppm)	[43]
	Phenanthrene			16–54 (200 ppm)	
*Bacillus licheniformis* MTCC 5514	Anthracene	22	100–1000	95 (300 ppm)	[25]
*Aeribacillus pallidus* strain SL-1	Naphthalene	5	100–1000	84 (1000 ppm)	[27]
	Phenanthrene			80 (200 ppm)	
*P. aeruginosa* NG4	Anthrone	10	100–1000	41 (300 ppm)	**This study**
	Naphthalene			91 (300 ppm)	
	Anthracene			88 (300 ppm)	
	Phenanthrene			80 (300 ppm)	
	Anthraquinone			33 (300 ppm)	
*P. brassicacearum* strain MPDS	Naphthalene	7	50	Completely (50 ppm)	[44]
*P. stutzeri* P2	Phenanthrene	7	100–1000	98 (1000 ppm)	[45]

## Data Availability

The data presented in this study are available in this article.

## Supplementary Materials

The following supporting information can be downloaded at: https://www.mdpi.com/article/10.3390/jox15010031/s1, Figure S1: Calibration curve of polyaromatic hydrocarbons used in this study. (A) Anthracene, (B) Anthrone, (C) Anthraquinone, (D) Phenanthrene, (E) Naphthalene; Figure S2: Primary characterization for BS production by *P. aeruginosa* NG4 under different concentrations of PAH: (A) Oil-displacement assay, (B) Drop collapse assay, (C) Emulsion Index; Figure S3: Sensitivity of *P. aeruginosa* strain NG4 against common antibiotics; Table S1: Sensitivity of *P. aeruginosa* strain NG4 against common antibiotics.
