# Supplementary material for: Degradation of Polyaromatic Hydrocarbons by Biosurfactant-Producing Pseudomonas aeruginosa NG4
Source: J Xenobiot. 2025 Feb 12;15(1):31. doi: 10.3390/jox15010031 (PMC11856512; doi:10.3390/jox15010031)
Supplement: Supplementary file 1 [file jox-15-00031-s001.zip › jox-3386837-supplementary.pdf]

Supplementary Information:

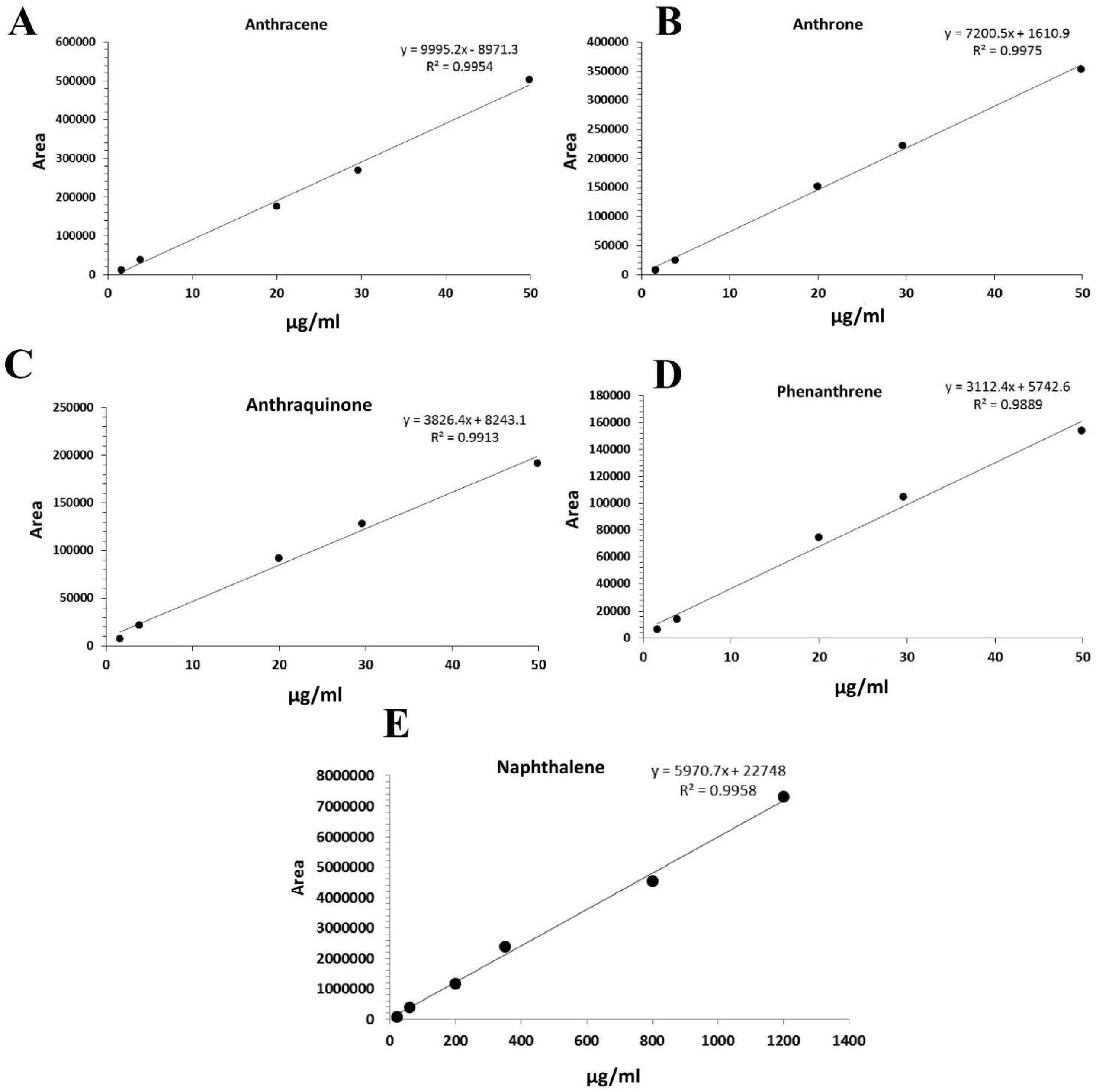

Figure S1. Calibration curve of polyaromatic hydrocarbons used in this study. (A) Anthracene, (B) Anthrone, (C) Anthraquinone, (D) Phenanthrene, (E) Naphthalene.

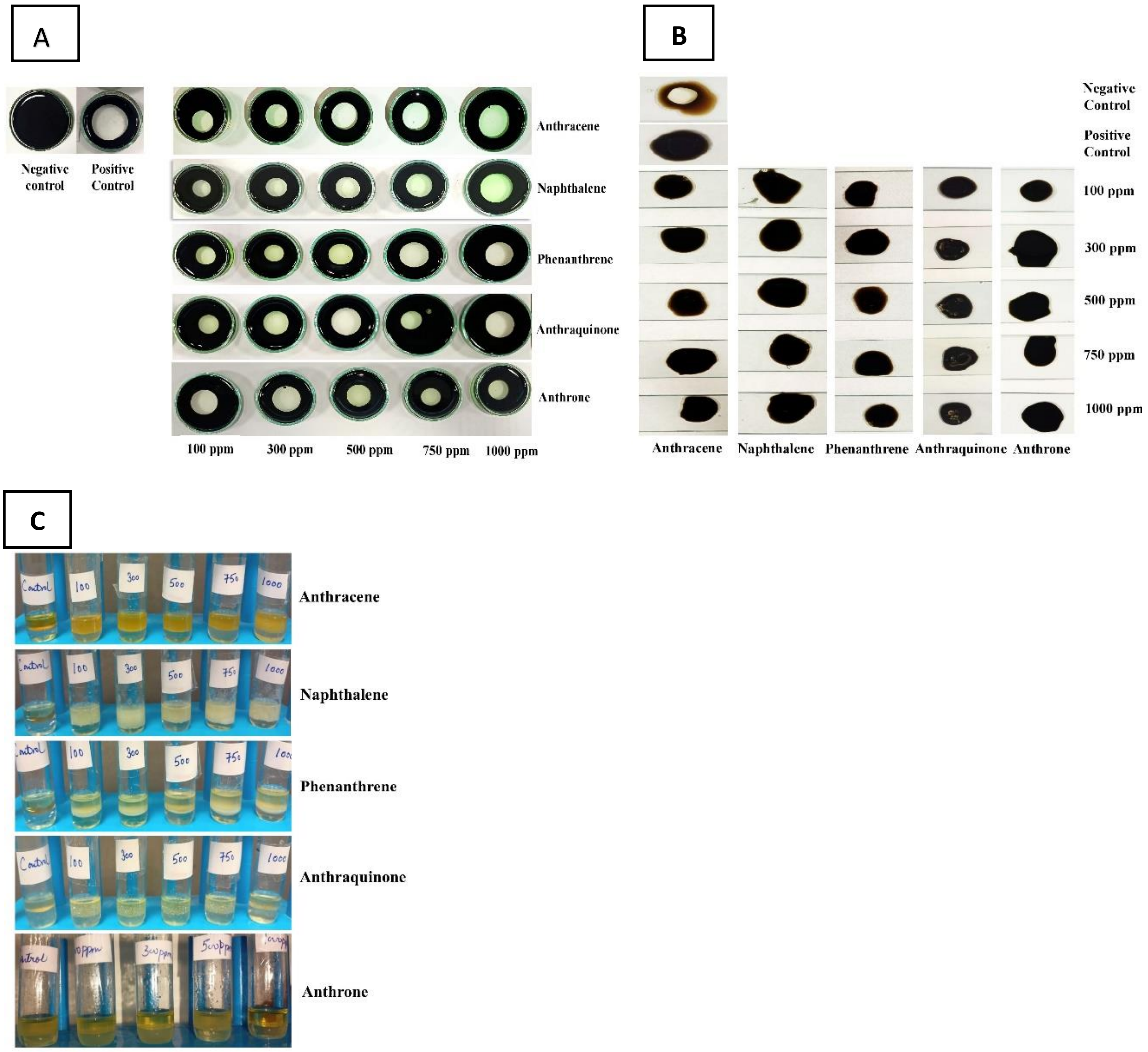

Figure S2. Primary characterization for BS production by *P. aeruginosa* NG4 under different concentrations of PAH A) Oil-displacement assay B) Drop collapse assay C) Emulsion Index.

Table S1. Sensitivity of *P. aeruginosa* strain NG4 against common antibiotics.

Antibiotic	Dose (μg)	Average diameter of bacteriostatic circle (cm)	Antibiotic resistance
Streptomycin	10	1.6±0.15	S
Ampicillin	10	-	R
Vancomycin	30	1.4±0.20	S
Tetracycline	30	1.7±0.10	S
Chloramphenicol	30	2.0±0.25	S

R: resistance, S: sensitivity

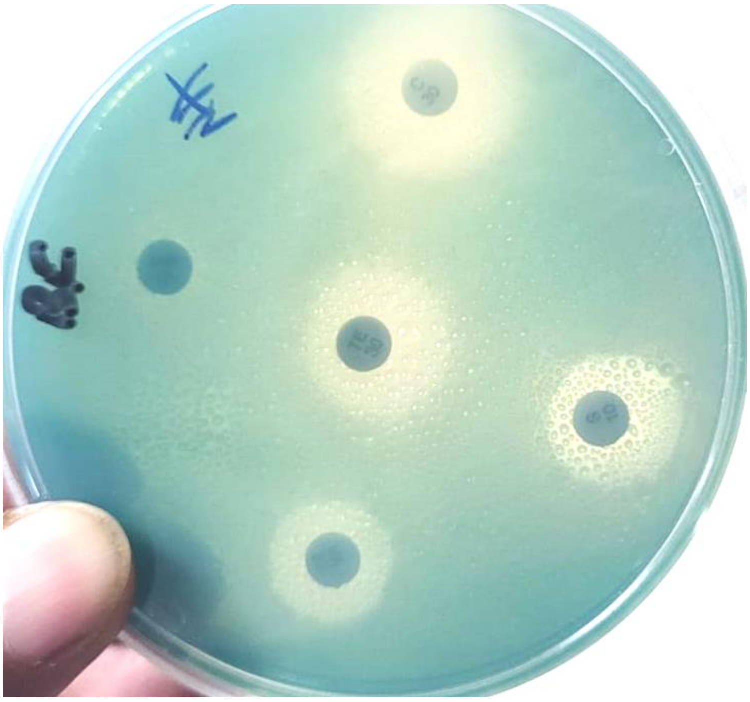

Figure S3. Sensitivity of *P. aeruginosa* strain NG4 against common antibiotics.
